# A Survey on Security Threats and Mitigation Mechanisms for Smart Hospitals in the 6G Era

**DOI:** 10.3390/s26134304

**Published:** 2026-07-07

**Authors:** Orestis Maraziotis, Georgios Mantas, Jonathan Rodriguez, Felipe Gil-Castiñeira

**Affiliations:** 1 Information Technologies Group, atlanTTic Research Center, University of Vigo, 36310 Vigo, Spain; xil@gti.uvigo.es; 2 Instituto de Telecomunicações, 3810-193 Aveiro, Portugal; gimantas@av.it.pt; 3 Faculty of Computing, Engineering and Science, University of South Wales, Pontypridd CF37 1DL, UK; jonathan.rodriguez@southwales.ac.uk

**Keywords:** 6G, edge networks, smart hospitals, Internet of Medical Things (IoMT), security and privacy, cybersecurity threats, security mechanisms, privacy-preserving technologies

## Abstract

Smart Hospitals integrated within 6G edge networks aim to enhance hospital connectivity and operational efficiency by enabling intelligent and personalized e-health services and applications while optimizing resource utilization and maintaining a high degree of autonomy. Nevertheless, the interconnectivity and 6G integration, which comprise core components of Smart Hospitals, are susceptible to a wide range of security threats, posing significant risks to the confidentiality, integrity, and availability of hospital data and operations. Given that security is a critical concern for Smart Hospitals, there is an urgent need to develop novel security mechanisms to safeguard these environments within 6G edge networks. In particular, this work highlights how defining 6G characteristics, such as Ultra-Reliable Low-Latency Communications, massive IoMT connectivity, distributed edge intelligence, and AI-native network operation, not only enable next-generation hospital services but also reshape the security and privacy threat landscape and the requirements of mitigation mechanisms. In this context, the first essential step is to comprehensively understand both existing and emerging threats targeting Smart Hospitals in the 6G edge network ecosystem. Therefore, this article provides a categorization of security and privacy attacks based on their primary targets. Moreover, it presents a survey of mitigation techniques derived from recent literature, specifically designed to counter threats facing Smart Hospitals in 6G edge networks. The intent is to establish a foundation that supports ongoing research towards the development of effective, 6G-aware security countermeasures capable of protecting Smart Hospitals under the stringent latency, scalability, and reliability requirements of future healthcare environments.

## 1. Introduction

Smart Health is an emerging paradigm that integrates advanced Information and Communication Technologies (ICT), the Internet of Medical Things (IoMT), Artificial Intelligence (AI), and next-generation networks to transform healthcare delivery, monitoring, and management [1,2]. Smart Hospitals, also known as Intelligent or Digital Hospitals, constitute a critical component of the smart health paradigm, incorporating cutting-edge technologies, intelligent systems, and data-driven approaches to optimize resource utilization, improve operational efficiency, and deliver patient-centered, next-generation healthcare services [3,4].

The concept of Smart Hospital first appeared in the late 1980s, initially focusing on intelligent buildings and workflow automation. Since the mid-2000s, advances in IoT, eHealth, and data-driven technologies have evolved this idea into a comprehensive healthcare model aimed at enhancing efficiency, patient care, and resource management [5,6]. According to the European Union Agency for Cybersecurity (ENISA), a Smart Hospital is defined as “a hospital that relies on optimized and automated processes built on an ICT environment of interconnected assets, particularly based on Internet of Things (IoT), to improve existing patient care procedures and introduce new capabilities” [7]. Additionally, the authors of [8] provide a more detailed definition of Smart Hospital which can be summarized as: “a healthcare environment with interconnected systems employing technologies such as IoMT, robotics, and mobile tools to collect data, automate processes, and generate insights”. These capabilities support enhanced decision-making, error reduction, increased patient involvement, resource optimization, improved staff performance, and cost reduction, ultimately elevating care quality and meeting rising healthcare demands. Furthermore, while Smart Hospitals hold significant potential, their implementation requires novel infrastructure and thoughtful consideration of emerging challenges, particularly those related to security and privacy within increasingly connected healthcare ecosystems. Key enabling technologies include Ultra-Dense Networks (UDNs), 6G networks, edge computing, and AI.

Massive connectivity is fundamental to Smart Hospital infrastructure. Sensors, actuators, medical equipment, mobile and wearable devices, implantable devices, identification systems, networking equipment, data storage solutions and facility management systems are all interconnected, continuously exchanging data. To support this, UDNs [9,10] are emerging as a key enabler for the demanding connectivity requirements of 6G. According to [9], a UDN is defined as a network where the number of access points or base stations within a given area (spatial density) is equal to or greater than the number of active end devices, such as user equipment or physical devices. UDNs significantly enhance network capacity, reduce latency, and improve energy efficiency—capabilities essential for seamless connectivity in Smart Hospitals. The deployment of multiple closely spaced small cells in 6G, combined with Massive-Input Multiple-Output (MIMO) systems, further supports these performance and capacity demands [4].

6G will serve as the backbone of connectivity in Smart Hospitals, enabling energy-efficient and sustainable systems [11]. Leveraging edge computing and AI, 6G networks will deliver ultra-high data rates, Ultra-Reliable Low-Latency Communications (URLLC), real-time decision-making, and optimized resource management [12]. In addition, advanced technologies such as beamforming, non-orthogonal multiple access (NOMA), massive MIMO, device-to-device (D2D) communication, and simultaneous wireless information and power transfer (SWIPT) will further enhance connectivity, efficiency, and the overall performance of Smart Hospitals’ networks [4,12,13]. Building on these capabilities, 6G introduces several defining features for Smart Hospitals, including massive IoMT device connectivity, ultra-dense small-cell and UDN deployments, network slicing, integrated sensing and communication, and pervasive edge intelligence. These features enable advanced applications such as immersive telesurgery, real-time patient and asset Digital Twins (DTs), large-scale remote monitoring, and extended reality (XR)-based clinical training, but they also significantly expand the cyber-physical attack surface and create new security and privacy risks that are not adequately captured by pre-6G threat models.

Equally important, AI and Machine Learning (ML) are integral to Smart Hospitals operating over 6G networks by enabling intelligent resource management, fostering network self-optimization and autonomy, enhancing operational workflows, and enabling personalized patient care [2,4,12,14]. The massive volume of heterogeneous healthcare data required to enhance patient care can only be effectively processed through AI capabilities. As detailed in [15] advanced ML, Deep Learning (DL), and Natural Language Processing (NLP) algorithms extract valuable insights from structured and unstructured sources including patient records, medical images, and clinical notes. These insights can be applied to predict diseases, detect outbreaks and pandemics, support accurate diagnoses, and identify anomalies in complex patterns that may indicate health issues [16]. In addition, AI systems uncover hidden correlations and nonlinear relationships across diverse datasets, enabling more precise predictions and tailored clinical recommendations [16]. Moreover, through continuous learning, AI models personalize treatment recommendations, ultimately improving patient outcomes [17]. Simultaneously, AI can optimize workflows and resource allocation, enabling healthcare organizations to operate more efficiently and deliver higher-quality patient care [14].

However, healthcare is a highly attractive target for adversaries because of its critical services, sensitive data, and complex interconnected systems. Smart Hospitals, in particular, are especially vulnerable due to their extensive connectivity and reliance on advanced technologies. The massive connectivity enabled by 6G increases security and privacy vulnerabilities [18], especially as intelligence shifts toward edge computing, where AI and computing algorithms run directly on distributed devices, facilitating autonomy [19]. While 6G supports connectivity for a vast number of edge devices and employs edge architectures that enable localized processing near end users—thereby reducing latency and minimizing unnecessary data transmission—it also introduces new security and privacy challenges. Additionally, as AI becomes an integral and inseparable component of 6G, inherent weaknesses in ML algorithms can be exploited through Adversarial Machine Learning (AML) techniques, further increasing the overall network risk. More specifically, the combination of massive IoMT connectivity, distributed edge intelligence, and 6G-native networking functions such as slicing and D2D communication broadens the attack surface across devices, data flows, learning models, and control mechanisms. At the same time, the stringent latency and reliability constraints of clinical services limit the applicability of many conventional security solutions, making it necessary to examine threats and defenses in direct relation to 6G architectural characteristics.

Although some studies have explored the use of 6G in healthcare [19,20], including Smart Hospitals [4], they mostly focus on general architecture and enabling technologies like IoT, AI, and robotics. For example, while the authors in [4] explore a 6G Smart Hospital model and identify security and privacy as important challenges, they leave the detailed analysis of specific threats and mitigation mechanisms for future work. To the best of our knowledge, the current literature has not deeply examined the specific security and privacy risks of 6G-enabled Smart Hospitals. Therefore, this work aims to advance the development of 6G-enabled Smart Hospitals by examining how 6G edge architectures, URLLC, massive IoMT connectivity, and edge AI reshape both the security and privacy threat landscape and the design of mitigation mechanisms. In this context, our contributions include a detailed review of security and privacy challenges in Smart Hospitals connected through 6G edge networks, along with an analysis of mitigation mechanisms targeting these issues. Specifically, the article provides a target-centric categorization of attacks relevant to Smart Hospitals operating over 6G edge networks and surveys privacy-preserving technologies and security mechanisms that are aligned with the performance, scalability, and architectural constraints of future 6G-enabled healthcare environments.

To ensure a transparent and reproducible review, we conducted a structured literature search. Relevant articles were collected exclusively from the IEEE Xplore and Google Scholar databases. Our search strategy was tailored to each section of the survey, using combinations of primary keywords such as “6G”, “Smart Hospital”, “IoMT”, “Security”, “Privacy”, and “Edge Computing”. To comprehensively investigate the threat landscape, we also used targeted keywords for specific attack vectors (e.g., “Data Poisoning”, “Evasion Attacks”, “Ransomware”, “DDoS”, “Man-in-the-Middle”, “IoT Botnets”, and “Digital Twin Manipulation”). In addition, we used targeted keywords for security mechanisms and privacy-preserving technologies commonly discussed in the literature (e.g., “Anomaly Detection”, “Zero Trust”, “SIEM”, “SOAR”, “Federated Learning”, “Compressive Sensing”, “Differential Privacy”, “Homomorphic Encryption”, and “Secure Multi-Party Computation”). We included peer-reviewed journal articles, conference papers, and formal technical reports. To capture the most recent technological advancements, our primary inclusion criteria focused on papers published between 2018 and 2026, although selected foundational security works were retained to provide the necessary theoretical context.

## 2. Architecture of Smart Hospitals in the 6G Edge Network

The transition from conventional healthcare facilities to Smart Hospitals is driven by the increasing demand for more efficient, personalized, and data-driven medical services. Factors such as rising patient volumes, aging populations, the need for pandemic resilience, and advancements in medical technologies are pushing hospitals toward highly interconnected, sensor-rich, and AI-enabled infrastructures. Originally motivated by administrative needs and asset management, early Smart Hospitals focused on reducing costs and minimizing accidents through improved resource tracking and process automation. Early research emphasized smart building aspects, including distributed sensors and networks, to automate professional workflows and monitor patient locations within healthcare facilities. Nevertheless, with the widespread adoption of wearable health sensors, advancements in network infrastructure, and the rise in networked e-health services, the concept of Smart Hospital has evolved beyond physical boundaries into an integrated, technology-driven healthcare ecosystem. This new paradigm embeds “smartness” at the core of healthcare service delivery, significantly transforming how services are provided [4,5].

Additionally, the emergence of 6G, and more specifically UDNs, will accelerate this transformation by enabling ultra-low-latency communication, massive device connectivity, and seamless integration of AI and IoMT at the network edge. These capabilities will support real-time remote diagnostics, immersive telepresence for surgery, extensive patient monitoring, high-definition video streaming for telemedicine, continuous monitoring of patients’ vital signs, and predictive analytics, unlocking healthcare applications that are not feasible with current network technologies [4]. At the same time, these enabling characteristics also determine the architectural exposure of Smart Hospitals to cyber and privacy risks. In particular, the tight coupling among sensing, communication, edge intelligence, and clinical applications means that attacks targeting one layer can rapidly propagate across the rest of the system, making a 6G-aware architectural understanding essential for threat analysis.

**Figure 1:**
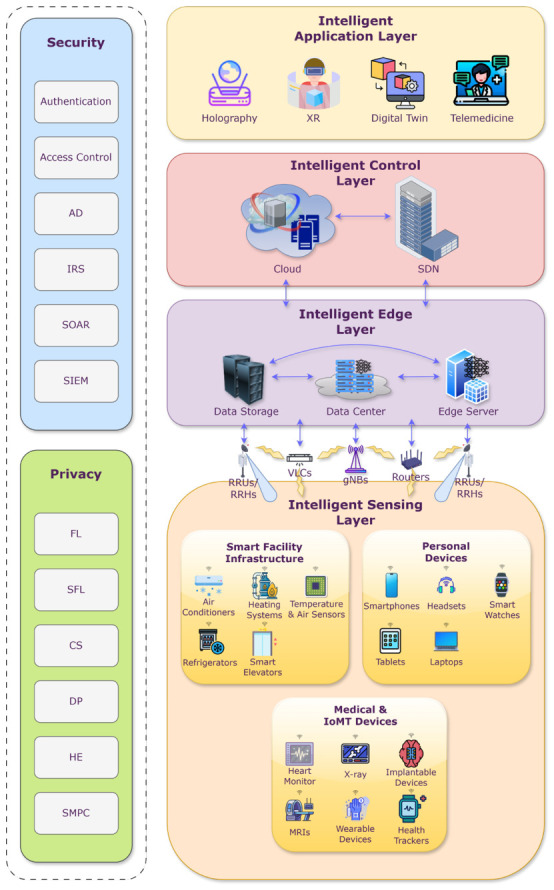
Architecture of a Smart Hospital in the 6G Edge Network.

In this section, we propose an architecture design for Smart Hospitals in the 6G edge network. The proposed design, illustrated in Figure 1, is based on the 6G architecture introduced by the authors of [21]. First, we present the four layers of this architecture, and then we provide envisioned deployment scenarios to demonstrate its feasibility and scalability, establishing the necessary context for the security and privacy threats detailed in Section 3.

### 2.1. Intelligent Sensing Layer

The Intelligent Sensing Layer forms the foundation of the Smart Hospital architecture and comprises interconnected medical devices, user devices, and smart facility infrastructure that continuously collect and transmit real-time data over the 6G edge network. Medical devices enable key Smart Hospital services such as telemedicine, Remote Patient Monitoring (RPM), automated medication delivery, and surgical assistance, increasing automation and enhancing decision-making capabilities [7]. These medical devices include: (i) Mobile devices (glucose, blood sugar measuring devices), (ii) Wearable devices (insulin pumps, temperature counters), (iii) Implantable devices (cardiac pacemakers), and (iv) Stationary devices (CT, MRI, X-ray).

Furthermore, in Smart Hospitals, user devices are intelligently integrated to deliver timely and context-aware information, facilitating mobility for staff and patients [7]. Examples include smartphones, laptops, tablets, smart watches, pagers, wearable health trackers, augmented reality (AR) headsets, handheld medical scanners, portable diagnostic tools, and voice-assisted communication devices. All these devices need to be seamlessly connected to the Smart Hospital’s network to communicate instantly and securely. When healthcare professionals have real-time access to patient and resource data, coordination improves and overall effectiveness in delivering care is significantly enhanced. Additionally, smart facility infrastructure comprises critical systems such as blood-product refrigerators, ventilation, power and climate control, temperature and humidity sensors, and medical gas supplies, ensuring safe and efficient hospital operations while accounting for staff, patient, and environmental factors. From a 6G perspective, this layer is not only the main source of real-time clinical and operational data but also the largest attack surface due to the scale, heterogeneity, and physical exposure of connected entities. The coexistence of implantable, wearable, stationary, and user-carried devices under massive 6G connectivity increases the likelihood of rogue device access, spoofing, data manipulation, and service disruption at the point of sensing.

### 2.2. Intelligent Edge Layer

The Intelligent Edge Layer serves as the intermediate processing tier in the Smart Hospital architecture, where data, Artificial Intelligence (AI), and fog computing converge to process information close to its source. This localized processing reduces latency, enhances data privacy, and enables real-time decision-making within the 6G-enabled Smart Hospital network. Connectivity and data transmission between devices of the Intelligent Sensing Layer and this edge layer are supported by the 6G edge network that integrates Remote Radio Units (RRUs), Remote Radio Heads (RRHs), routers, Next-Generation Node Bs (gNBs), and Visible Light Communication (VLC) technologies. Together, these components facilitate high-speed, low-latency communication across the hospital environment.

Edge computing, empowered by 6G networks, brings computing and storage resources closer to IoMT devices, enabling immediate data processing, analysis, and rapid responses for critical applications such as energy management and security. While edge resources reduce latency, their processing capacity is limited compared to centralized cloud servers. To address this, integrated edge-cloud architectures are deployed to balance performance, scalability, and responsiveness for time-sensitive or resource-demanding applications [22,23]. This layer is particularly critical in 6G-enabled Smart Hospitals because latency-sensitive services increasingly depend on localized inference, filtering, orchestration, and security enforcement at the edge. As a result, edge nodes become high-value targets for adversaries seeking to tamper with medical data streams, compromise AI models, or disrupt time-critical services before information reaches centralized control systems.

Data is among the most essential and sensitive assets of Smart Hospitals, including clinical and administrative patient information, staff records, financial documents, inventory logs, and research data [7]. Beyond supporting routine operations, this data supports critical functions such as disease prediction, outbreak detection, personalized treatment planning, and resource management [6]. By integrating and analyzing diverse datasets, Smart Hospitals optimize workflows, improve patient outcomes, and ensure efficient allocation of both medical and non-medical resources. Furthermore, AI plays a pivotal role by leveraging this rich data pool to enhance healthcare outcomes. It strengthens clinical decision support systems and augments human expertise through complex pattern recognition on large datasets beyond conventional analytical capabilities [6,24]. According to [25] AI use cases in healthcare are categorized into clinical decision making, hospital operations and management, medical imaging and diagnostics, and patient care and monitoring.

**Figure 2:**
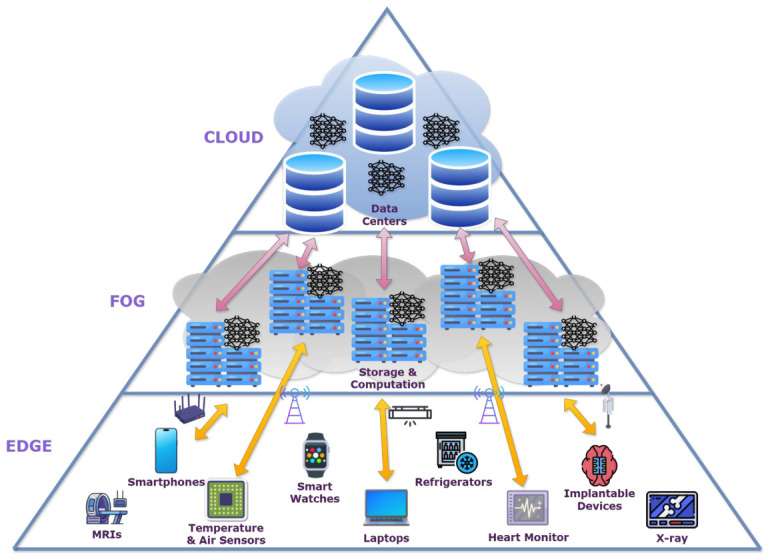
Cloud, Fog, and Edge connection.

Additionally, fog computing complements edge computing by performing processing and analytics closer to the network edge, acting as an intermediary between edge devices, such as IoT sensors or medical equipment, and centralized cloud servers [26,27]. This layered approach supports real-time decision-making, privacy-sensitive processing, and cost reduction by minimizing data transmission to centralized servers. Advances in portable devices, AI, and cloud technologies have established a strong foundation for IoT proliferation in healthcare, thus accelerating the development of Smart Hospitals and transforming many aspects of patient care [28]. Figure 2 illustrates the interconnection between cloud, fog, and edge computing layers. Accordingly, the Intelligent Edge Layer should be interpreted not only as a performance enabler but also as a primary security boundary in the proposed architecture, since it mediates between constrained sensing devices, distributed analytics, and higher-layer decision-making components.

### 2.3. Intelligent Control Layer

The Intelligent Control Layer is the central management and decision-making tier in the Smart Hospital architecture within the 6G edge network. It integrates cloud computing, AI, and Software-Defined Networking (SDN) to provide centralized control, dynamic network management, and intelligent orchestration of resources. This layer processes and optimizes data collected from the Intelligent Sensing and Intelligent Edge Layers, ensuring efficient routing, reliable connectivity, scalability, and adaptive network behavior throughout the Smart Hospital network.

Cloud computing offers scalable services such as servers, databases, and software platforms that support rapid deployment and flexible resource allocation. Within healthcare, cloud infrastructure enables the storage and processing of vast amounts of clinical, administrative, and operational data, as well as running compute-intensive applications without dependence on local hardware [28]. In addition, AI is a crucial enabler in the Intelligent Control Layer, sustaining high Key Performance Indicators (KPIs) such as low latency, throughput, and reliability while efficiently managing resources [12]. As a key enabling technology, AI facilitates self-sustainable, zero-touch networks capable of optimizing handovers, reducing energy consumption, autonomously detecting, predicting, and mitigating network anomalies [20]. This intelligent automation supports the complex and dynamic requirements of Smart Hospitals, ensuring seamless and secure communication across distributed systems. Because this layer coordinates orchestration, policy enforcement, and adaptive network behavior, it also becomes a strategic target for attacks against trust, availability, and control integrity in 6G-enabled Smart Hospitals. Compromise at this layer can have system-wide effects, including misallocation of network resources, unsafe automation decisions, and cascading disruptions across clinical and non-clinical services.

### 2.4. Intelligent Application Layer

The Intelligent Application Layer is the user-facing tier of the 6G-enabled Smart Hospital architecture, responsible for delivering advanced healthcare services and applications. It builds upon the computational power and connectivity provided by the underlying layers to enable innovative healthcare applications. Key applications supported by this layer include holographic communication and training, XR for remote surgeries and educational purposes, Digital Twins (DTs) for real-time monitoring of patients and hospital facilities, and telemedicine for seamless remote consultations [6,12,20]. By translating the network’s computational and analytical capabilities into practical solutions, this layer enhances both patient care quality and clinical operational efficiency. These applications are also the clearest illustration of why 6G matters in the Smart Hospital context: they depend on stringent communication and computation guarantees, yet any disruption, manipulation, or privacy breach at this layer can translate directly into degraded care quality, unsafe clinical decisions, or exposure of highly sensitive patient information.

### 2.5. Illustrative Practical Deployment Scenarios

To illustrate the feasibility and scalability of the proposed architecture in practical 6G-enabled Smart Hospital settings, we present two envisioned deployment scenarios. As 6G networks remain in the standardization and pre-deployment phases, empirical evidence from real-world 6G systems is not yet available, and current research is therefore theoretical, simulation-based, or exploratory [4,19,20]. Consequently, the following scenarios illustrate how the proposed architectural layers could interact to support 6G-enabled Smart Hospitals, while highlighting the specific security and privacy challenges to the 6G era. These scenarios are not included merely as application examples; they also serve to examine how specific 6G features create distinct operational and security dependencies. In both cases, the same mechanisms that enable low latency, synchronization, immersive interaction, and distributed intelligence also introduce new attack paths that must be considered when designing protections for Smart Hospitals.

#### 2.5.1. Scenario A: Immersive Telesurgery and Remote Support

In this scenario, a specialized remote surgeon uses immersive Augmented Reality (AR) headsets to provide real-time guidance to a local operating room team or to directly perform interventions via robotic assistance [29,30]. Such complex telesurgery operations rely heavily on 6G URLLC to seamlessly stream high-definition video and critical tactile feedback without perceptible delay [30,31]. To sustain the extreme performance requirements of these time-sensitive medical applications, our proposed architecture routes the workflow through its layers. The massive volume of operational data generated by the surgical equipment is aggregated and processed locally by nodes within the Intelligent Edge Layer, minimizing transmission latency and optimizing responsiveness. Thus, this highly optimized data stream enables the seamless, real-time execution of the AR guidance service, which is delivered to the surgeon through the Intelligent Application Layer.

However, the critical nature of this time-sensitive workflow makes it a high-value target for interception, manipulation, or disruption [18], directly endangering patient safety. This necessitates the deployment of advanced edge-based security mechanisms (e.g., Anomaly Detection (AD)) and architectures (e.g., Zero Trust Architecture (ZTA)), as detailed in Section 4, to ensure continuous, authenticated, integrity-preserving, and interference-resilient connectivity.

#### 2.5.2. Scenario B: Real-Time Patient Digital Twins (DTs)

Recent literature establishes that real-time healthcare DTs rely on continuous acquisition of physiological parameters, such as heart rate and oxygen saturation [32], to tightly couple a physical patient’s observable state with a precise digital replica [33]. This synchronization is critical for enabling AI-driven predictive analytics that autonomously detect clinical anomalies [32,34]. To implement this in a real-world Intensive Care Unit (ICU) scenario, our proposed 6G-enabled architecture distributes the DT lifecycle across its layers. Initially, a vast set of interconnected medical devices within the Intelligent Sensing Layer, such as implantable cardiac pacemakers and wearable sensors, continuously collect and transmit raw biometric data. Next, fog and edge computing nodes within the Intelligent Edge Layer aggregate and process this massive flow of data close to its source, leveraging localized processing to ensure that the patient’s real-time DT is continuously updated and hosted within the Intelligent Application Layer. Finally, this synchronized digital replica empowers AI components within the Intelligent Control Layer to perform centralized orchestration and predictive analytics, autonomously identifying potential health anomalies before they manifest clinically.

Nevertheless, the massive connectivity required by these diverse IoMT devices vastly expands the network’s attack surface, increasing susceptibility to rogue node activity, data manipulation, and DT synchronization attacks [18,19]. Furthermore, because the DT relies entirely on highly sensitive Protected Health Information (PHI), robust privacy-preserving technologies, such as Federated Learning (FL) and Differential Privacy (DP), alongside continuous model and state verification, should be deployed within the Intelligent Edge Layer. As detailed in Section 4, these privacy-preserving technologies and hardening mechanisms facilitate secure, decentralized AI operations over 6G-enabled Smart Hospital infrastructures.

## 3. Threat Landscape Overview

The healthcare sector reports the highest number of security breaches and data privacy violations. In Smart Hospitals, the extensive interconnection of user and medical devices, combined with the handling of highly sensitive data, makes them prime targets for malware attacks and unauthorized intrusions. These attacks often aim for financial gain or theft of private information, while simultaneously compromising patient health and safety [35,36]. Moreover, many IoMT devices deployed in Smart Hospitals are designed with energy efficiency as a priority rather than security, leading to critical vulnerabilities exploitable by malicious actors. The integration of AI introduces additional threats, potentially compromising diagnoses and exposing sensitive patient data. Furthermore, reliance on 6G edge networks, with their massive device connectivity and distributed processing capabilities, significantly expands the attack surface, increasing risks to data security, patient privacy, and service availability. This section examines the evolving threat landscape on Smart Hospitals operating within 6G edge networks. However, due to the limited availability of direct research on Smart Hospitals and their specific threats, insights are primarily drawn from research works related to the broader domains of IoMT-based smart healthcare, hospital cybersecurity, and 6G security and privacy. For this reason, the threat landscape in Smart Hospitals should not be viewed as a simple extension of conventional hospital cybersecurity. Rather, it reflects the convergence of cyber-physical healthcare risks with 6G-specific characteristics such as AI-native operation, dense edge deployment, ultra-low-latency communications, and large-scale heterogeneous device participation.

### 3.1. Threats to Smart Hospitals in 6G Edge Networks

To understand the threats faced by Smart Hospitals in 6G edge networks, attacks are systematically categorized based on their primary target and mapped to the architectural layers described in Figure 1. This structured classification distinguishes four main categories: (i) AI threats, (ii) network threats, (iii) medical and personal devices threats, and (iv) human-centric threats. Such a target-based categorization facilitates a comprehensive assessment of the security measures necessary for each layer. Table 1 presents an overview of these threats, indicating their primary (✓) and secondary (✕) targets. Furthermore, the table presents the main attack vectors used to execute each threat and identifies which core security goals (i.e., Confidentiality, Integrity, and Availability) are compromised.

While existing literature typically categorizes threats by architectural layer (e.g., physical, link, or service) [30], by compromised security objectives (e.g., Confidentiality, Integrity, and Availability) [37], or by system openness and isolated technology-specific vulnerabilities [38], the differentiation of our proposed classification lies in its target-centric methodology. Whereas aligned with the architecture from [21], our classification organizes threats by their primary targets to more accurately capture the multi-dimensional attack surface of a 6G-enabled Smart Hospital. This target-centric structure is particularly suitable for 6G-enabled Smart Hospitals because threats in these environments often propagate across multiple layers simultaneously. For example, a compromise originating at the device or data level may rapidly affect edge intelligence, network orchestration, and user-facing clinical applications, making purely layer-isolated classifications less expressive for this setting.

#### 3.1.1. AI Threats

AI in Smart Hospitals operating over 6G edge networks faces a range of sophisticated threats that can compromise patient data confidentiality, undermine model integrity, and adversely affect clinical decision-making. AI components are primarily embedded within the Intelligent Edge Layer and the Intelligent Control Layer of the proposed architecture, where data processing and decision-making occur.

Key security threats include data poisoning attacks (data injection, data manipulation, and logic corruption), where malicious entities tamper with training data by injecting carefully crafted false samples. This manipulation can distort the ML system’s training phase, leading to inaccurate learning and erroneous decisions, which may negatively impact patient care [18,21,36,39,40]. The distributed nature of smart healthcare systems further amplifies vulnerability to such attacks, as noted by [41]. Moreover, membership inference exploits ML models to determine whether specific data points were included in the training dataset, potentially exposing sensitive information. Conversely, reverse membership inference seeks to identify data points excluded from training, indirectly revealing confidential data [21,42]. Furthermore, model inference, including model stealing (extraction) and model inversion, allows attackers to probe AI models. Model stealing exposes the model’s architecture and parameters, enabling unauthorized replication, while model inversion reconstructs sensitive training data, risking privacy violations [18,21,41,43]. Additionally, evasion occurs during the testing phase, wherein maliciously altered input data is used to circumvent detection and degrade the model’s performance [18,21,40,43].

To illustrate these threats in a clinical context, an adversary can deploy imperceptible AML techniques to alter the pixel data of medical images (e.g., X-rays, MRIs, or CT scans), acting as targeted implementation methods that exploit Convolutional Neural Networks (CNNs) to degrade diagnostic performance [44,45]. In a 6G-enabled Smart Hospital setting, these unmitigated degradations could result in an edge-hosted diagnostic AI model misidentifying a malignant tumor as benign, leading doctors to underestimate disease severity and ultimately create serious dangers for patients [45,46]. Similarly, poisoning attacks can inject manipulated biometric thresholds into the training data of localized edge nodes, systematically degrading the accuracy of autonomous patient monitoring systems over time [47,48].

Beyond these conventional AI threats, the integration of Generative AI (GenAI) into 6G networks introduces a highly sophisticated new attack surface. While GenAI models offer considerable benefits for network management through their ability to learn complex data distributions, they simultaneously empower adversaries with sophisticated offensive capabilities. Specifically, these models can be exploited to generate synthetic data that realistically mimics clinical datasets, enabling the construction of realistic adversarial samples that corrupt diagnostic pipelines or evade wireless security frameworks. Additionally, GenAI models can automate highly convincing, personalized spear-phishing campaigns targeting hospital staff with privileged access, directly exploiting the human element of the classification [49].

Alongside GenAI, the deployment of AI-driven DTs represents another highly critical, emerging attack surface. In a 6G-enabled Smart Hospital environment, these DTs act as highly synchronized, real-time virtual counterparts of physical patients, medical devices, and hospital assets. Leveraging 6G URLLC, these DTs are continuously updated, which means that data manipulation or synchronization attacks targeting the AI models and data flows can translate into immediate physical risk. If an adversary corrupts the continuous data stream between the physical entity and its digital replica, or exploits vulnerabilities within the virtual environment, the system may fail to identify clinical or device anomalies in a timely manner. Consequently, a corrupted DT may feed faulty data to the Intelligent Edge Layer, causing automated edge systems to incorrectly adjust critical life-support parameters, smart facility ventilation settings, or automated drug delivery dosages, with potentially severe consequences for patient safety [50]. The relevance of these threats is amplified in 6G-enabled Smart Hospitals because AI is not a peripheral tool but a native operational component of both communication and clinical workflows. Consequently, attacks on AI models may affect not only prediction quality but also scheduling, orchestration, AD, and automated response functions that sustain critical hospital services.

Despite the growing body of literature addressing AI security threats within 6G networks, there remains a notable gap in research specifically addressing AI vulnerabilities in Smart Hospitals leveraging 6G edge networks. Given the critical role of AI in analyzing sensitive healthcare data, supporting clinical decision-making, and enabling real-time healthcare services, this underexplored area presents substantial risks if not adequately studied and mitigated. Consequently, comprehensive investigations into the unique challenges, threat models, and defense mechanisms for AI systems in 6G-enabled Smart Hospitals are urgently needed to ensure secure, reliable, and privacy-preserving healthcare delivery.

#### 3.1.2. Network Threats

The network infrastructure in Smart Hospitals operating over 6G edge networks represents a broad surface of multiple risks. To systematically analyze these risks, threats have been categorized into two primary domains based on their key targets: core computer networks and IoMT networks. This distinction facilitates a more granular understanding of the security challenges unique to each domain and supports targeted mitigation strategies. In the 6G setting, these network threats must also be interpreted in relation to new architectural features, including ultra-dense deployments, D2D communication, software-defined control, and highly distributed edge processing. These characteristics improve responsiveness and scalability, but they also multiply communication paths, trust dependencies, and opportunities for stealthy disruption or interception.

##### Computer Network Threats

The computer network in Smart Hospitals operating within 6G edge networks encompasses several critical components. These include the core IT infrastructure, which comprises data centers, application servers, and edge servers, as well as the communication backbone, including LAN, VLAN, Wi-Fi, 6G core connectivity, and VLC. Additionally, the network integrates comprehensive security and control systems, including firewalls, IDS/IPS, VPNs, authorization frameworks, identity and access management, staff management, auditing and logging mechanisms. According to the proposed architecture in Figure 1, these components primarily reside within the Intelligent Edge Layer and the Intelligent Control Layer, providing essential processing, management, and security capabilities at the network edge and control planes.

A significant security threat to the computer network in Smart Hospitals is eavesdropping, where communications are intercepted and monitored to access private information, such as patient records or authentication credentials, without disrupting normal network operations. This category includes packet sniffing, Man-in-the-Middle (MitM), replay, traffic analysis, session hijacking, and parallel session threats [18,36,39,41,51,52]. Due to its physical characteristics, VLC is particularly susceptible to such interception threats [21]. Additionally, Denial-of-Service (DoS) and Distributed-DoS (DDoS) attacks threaten the availability of servers, applications and communication channels by flooding them with excessive or malicious traffic, rendering them unavailable to legitimate users. In the context of Smart Hospitals, these attacks can disrupt access to critical systems/platforms such as Electronic Health Record (EHR) systems and telemedicine platforms [18,39,40,43,51,52].

Furthermore, another critical threat to the computer networks in Smart Hospitals is forgery, which involves the creation, alteration, or impersonation of data or system identities to deceive systems or users into granting unauthorized access [42]. Common techniques include spoofing, where a device or system identity is falsified; impersonation, where an entity pretends to be a legitimate user; and masquerading, where an attacker poses as a trusted identity to perform unauthorized actions. These forgery threats can severely compromise authentication credentials, system integrity, and the confidentiality of sensitive patient information [41].

Moreover, malware threats, including trojans, spyware, viruses, and ransomware, pose substantial risks by potentially damaging, disrupting, or gaining unauthorized access to critical healthcare information [18,39,40,52]. Among these, ransomware poses a particularly severe threat in healthcare environments due to its ability to encrypt critical clinical and operational data, effectively blocking access until a ransom is paid. Such disruptions can critically impact patients’ safety and hospital functionality, underscoring the urgent need for robust prevention and response mechanisms [35,38,51,53,54,55].

Other notable network threats include brute force attempts targeting authentication credentials by repeatedly guessing passwords, SQL injection compromising databases through malicious code, cross-site scripting (XSS) that injects malicious scripts into web applications to exfiltrate data or hijack sessions, and cookie manipulation where session cookies are altered or stolen to impersonate users [51]. These threats can lead to unauthorized access, data breaches, and significant disruption of healthcare services.

##### IoMT Network Threats

The IoMT network in Smart Hospitals within 6G edge environments constitutes the communication backbone that interconnects medical and personal devices. This network includes short-range wireless protocols, IoT gateways, and 6G-enabled devices, as well as security and control mechanisms such as device authentication, secure data transmission, and intrusion detection systems to protect connected medical devices. Within the proposed architecture, shown in Figure 1, the IoMT network primarily operates across the Intelligent Sensing Layer and the Intelligent Edge Layer.

Key security threats to the IoMT network involve disruptions to the availability and integrity of connected devices and data transmissions. These include threats analogous to DoS and DDoS scenarios, exemplified by wormhole, blackhole, collision, congestion, HELLO flood, and jamming conditions. Such disruptions can impair critical medical devices and sensors, delay or obstruct patient data transmission, and interfere with real-time monitoring and treatment processes [18,39,40,43,51,52,56]. Additional threats include byzantine behaviors, where compromised nodes act maliciously or unpredictably by sending false or inconsistent information, thereby degrading IoMT data integrity, routing accuracy, and decision-making reliability [36,39,41]. Routing threats arise when malicious nodes manipulate data packet paths, disrupting normal communication flows [51]. Shilling threats occur when malicious nodes inject fabricated data or reports that influence collaborative systems such as recommender systems, data aggregation, or routing decisions [36]. Furthermore, desynchronization threats target the timing or coordination of network communications, causing devices to lose synchronization [51]. Unauthorized node injection introduces rogue nodes into the network, while node subversion compromises existing nodes, both enabling malicious activities that disrupt communications and data integrity within IoMT networks [51,52]. In addition, sybil threats involve a single node creating multiple fake identities to gain disproportionate influence, manipulate routing, or undermine trust and data aggregation processes in IoMT networks [18,39,52]. Finally, 6G-enabled IoMT infrastructures must also account for emerging quantum-era attacks that could undermine public-key cryptography used for device authentication and secure channels, motivating a transition to quantum-safe schemes in future deployments [57]. These risks are especially consequential in Smart Hospitals because IoMT communication is tightly coupled with patient monitoring, treatment continuity, and automated clinical support. In a 6G environment, failures in trust establishment or routing integrity can therefore escalate quickly from network anomalies to patient-safety.

#### 3.1.3. Device Threats

The devices within Smart Hospitals operating in 6G edge networks create a highly interconnected environment, enabled by the integration of IoMT and ultra-reliable 6G connectivity [4,58]. While this interconnectivity is essential for seamless patient monitoring and efficient medical operations, it also exposes the system to multiple vulnerabilities. To effectively evaluate these risks, device-related threats are categorized based on their primary targets into medical devices and personal devices. Both categories of devices are integrated in the Intelligent Sensing Layer of the proposed architecture, as shown in Figure 1. This placement underscores their essential role in data acquisition and environmental sensing, serving as the primary interface for collecting critical health-related information within the Smart Hospital ecosystem. This device-centric exposure becomes even more pronounced in 6G-enabled settings, where a much larger number of heterogeneous endpoints participate continuously in sensing, communication, and actuation. As a result, device compromise should be viewed not only as an endpoint problem but as a systemic threat with potential consequences for edge analytics, DTs, and real-time medical workflows.

##### Medical Devices Threats

Medical devices within 6G-enabled Smart Hospitals are highly interconnected through IoMT, making them critical targets for threats that could directly compromise patient safety and quality of care. With the large number of medical devices connected in such environments, threats such as DoS attacks and DDoS attacks can be amplified, significantly impacting hospital operations [21,51,52]. IoT-botnet attacks, which involve compromising numerous IoMT devices such as sensors or monitors, can establish a botnet under an attacker’s control. These botnets are often exploited to launch large-scale DDoS attacks, disrupt hospital services, or spread malware across the network [21,52,59,60]. Additionally, tampering attacks, involving unauthorized modification of device hardware, firmware, or data, pose another serious risk, especially ones that can be executed physically or remotely, such as corrupting firmware or falsifying sensor readings. Such modifications can lead to incorrect diagnoses, inappropriate treatments, and data integrity breaches, ultimately endangering patient safety [18,39,52].

Moreover, firmware modification threats target non-volatile code, which controls hardware functionality—this is especially critical in wearable and implantable devices. Adversaries exploit firmware update mechanisms, reverse engineer communication protocols, or analyze application code to replace legitimate firmware with malicious versions, thereby gaining persistent control over the device [51]. Additionally, resource exhaustion threats, such as battery or energy drainage, force devices to perform excessive operations or communications, rapidly depleting batteries and rendering devices unavailable for legitimate users [52]. Finally, device cloning or replication threats involve adversaries extracting credentials or configuration data from legitimate sensors and using this information to create multiple cloned devices within the network. This undermines authentication mechanisms and security controls, facilitating further malicious activities [52].

##### Personal Devices Threats

The massive data stream of unmanaged personal devices, such as patient and visitor smartphones, smartwatches, laptops, and commercial mixed-reality headsets [61], represents a highly critical, yet underexplored, attack surface of 6G-enabled Smart Hospitals. Within the Intelligent Sensing Layer, these devices frequently operate in close physical and network proximity to critical IoMT systems, inadvertently creating an untrusted shadow network inside the 6G-enabled Smart Hospital environment [62]. Because 6G networks leverage D2D communication and localized edge computing [43], adversaries can exploit these unmanaged personal devices as low-security entry points to execute sophisticated lateral movement attacks. For instance, a compromised visitor smartphone could, in principle, bypass hospital perimeter defenses and act as a malicious intermediary node, launching localized DoS attacks against nearby medical monitors, and exploiting the cross-layer vulnerabilities inherent in wireless IoMT architectures [63]. Consequently, our review reveals the lack of sufficient literature addressing threats targeting personal devices within 6G-enabled Smart Hospitals, highlighting an urgent need for future research to formally model these attack paths and develop mitigation mechanisms that can safely integrate the inevitable presence of personal devices into the ecosystem without compromising the clinical infrastructure.

#### 3.1.4. Human-Centric Threats

Lastly, humans represent unpredictable and challenging targets in Smart Hospitals, as patients, medical personnel, and visitors can be manipulated or deceived by malicious actors to gain unauthorized access or compromise sensitive information. For example, keylogging techniques can capture a patient’s keystrokes during the entry of personal or medical data, allowing attackers to steal credentials and access sensitive health records. Similarly, phishing schemes deceive patients into submitting health information through fraudulent emails or deceptive links, which can subsequently be exploited for identity theft, financial fraud, or blackmail [51]. Given the critical role of human factors in healthcare cybersecurity, comprehensive training and continual awareness programs are essential to mitigate these threats. Despite advanced technological safeguards, the human element remains a significant vulnerability, necessitating ongoing education to empower all stakeholders to recognize and resist social engineering attempts, thereby safeguarding patient privacy and maintaining trust within healthcare systems.

## 4. Mitigation Mechanisms

Unlike financial data, stolen PHI cannot be changed, thus retaining long-term value for criminal activities such as identity theft and medical fraud. On the dark web, PHI is estimated to be up to 20 times more valuable than credit card or social security data [35], significantly amplifying the potential harm and severity of consequences following a data breach. Consequently, robust and efficient mitigation mechanisms are necessary to ensure the privacy and security of patients’ personal and medical information, as well as the sensitive data of Smart Hospitals. This section presents security and privacy countermeasures, drawn from the literature, aimed at addressing the various threats discussed previously in the context of Smart Hospitals operating within 6G edge networks. In 6G-enabled Smart Hospitals, these mitigation mechanisms cannot be considered in isolation from the underlying communication and edge architectures. Their design and deployment must explicitly account for 6G-specific characteristics such as ultra-dense and heterogeneous connectivity, distributed edge computation, AI-native control, and strict end-to-end latency guarantees, all of which constrain how security functions can be implemented and orchestrated in practice.

### 4.1. Security Mechanisms

#### 4.1.1. Authentication Mechanisms

Authentication is a fundamental security process that verifies the identity of users, devices, or systems to ensure that only legitimate entities participate in the trusted operations within Smart Hospitals [64]. A robust authentication mechanism is essential to prevent unauthorized access to personal computing devices (e.g., smartphones, watches, glasses) as well as critical Smart Hospital resources such as patient information, medical devices, and hospital infrastructure [65]. In the context of Smart Hospitals operating within 6G edge networks, strong authentication mechanisms ensure that only verified entities can interact with these resources, thereby mitigating risks to privacy, operational integrity, and overall security.

Authentication methods are generally categorized based on the factors used to verify identity. The main categories are as follows [56,64,66,67,68]: (i) knowledge-based, relying on information known only to the user, such as passwords or PINs; (ii) possession-based, utilizing items the user possesses, like smart cards, tokens, or digital certificates; (iii) biometric-based (inherence-based), verifying identity through unique physical traits such as fingerprints, iris scans, or facial recognition; (iv) location-based, granting access based on the user’s physical or network location; (v) behavior-based, leveraging patterns such as typing rhythm or gait; and (vi) adaptive risk-based, dynamically adjusting authentication requirements based on contextual risk factors. In 6G networks, additional authentication mechanisms become crucial to secure seamless access across heterogeneous devices, distributed IoMT systems, and multi-connectivity edge environments [69]. These mechanisms include mutual authentication, physical layer authentication, token-based authentication, handover authentication, and key agreement-based authentication.

Several recent works have proposed innovative authentication architectures tailored to healthcare and IoMT contexts. For instance, the authors of [65] introduced a Centerless User-Controlled Single Sign-On (CL-UCSSO) architecture combining smart card, password, and biometric-based three-factor authentication with time-bound properties. This approach offers fast, secure, and privacy-preserving access for patients and providers. The protocol’s security was formally verified using tools such as RoR [70,71], AVISPA [70,72], and BAN logic [73,74], and its performance evaluation showed improved functionality and reduced costs compared to prior works. Similarly, the authors of [75] proposed a cost-effective authentication solution optimized for 6G-enabled Artificial Intelligence of Medical Things (AIoMT) healthcare applications, designed for resource-constrained devices. Their protocol leverages lightweight cryptographic primitives and physically unclonable functions to secure communications against cyber and physical threats. It utilizes a Cloud of Things (CTS) server for secure key negotiation and demonstrates superior computation and communication efficiency compared to existing approaches. Moreover, given the integral role of fog computing in 6G-enabled Smart Hospitals, securing it and ensuring only authorized user access is paramount. Taking this into consideration, the authors of [76] proposed SAKA-FC, a new secure key management and user authentication scheme for fog computing environments. SAKA-FC employs lightweight operations suitable for resource-constrained smart devices, preserving anonymity and untraceability while imposing low communication and computation overheads. Its security has been validated using RoR, AVISPA, and informal security analysis [76].

Further, blockchain technology shows promise in enhancing security within IoMT edge networks. As highlighted in [37], blockchain can strengthen authentication and authorization and ensure tamper-proof data transmission. However, the authors note the lack of IoMT-specific solutions and call for efficient blockchain-based mechanisms adapted from broader IoT contexts. Addressing this gap, the authors of [77] proposed BTHRiD, a blockchain-based mechanism for trustworthy healthcare data sharing in 6G-IoT networks. While BTHRiD demonstrates strong performance in securing data sharing, it faces challenges such as the high computational demands placed on consensus nodes. To improve efficiency and scalability, the authors suggest leveraging techniques such as data compression and ML. Looking ahead, the long-term security of authentication in 6G-enabled Smart Hospitals must also account for emerging quantum-era threats, since large-scale quantum computers could break widely deployed public-key schemes used for device and user authentication. This motivates the gradual adoption and evaluation of quantum-safe (post-quantum) authentication and key-establishment protocols that can be implemented on resource-constrained IoMT devices without violating latency and energy constraints [78].

In conclusion, although numerous authentication schemes have been proposed for healthcare and IoMT-based systems, future research should focus on the development of lightweight and efficient mechanisms specifically tailored to meet the unique requirements of Smart Hospitals operating within 6G edge networks. Such mechanisms should balance strong security guarantees with the resource constraints of connected devices and the dynamic, heterogeneous nature of 6G-enabled medical environments.

#### 4.1.2. Access Control

Access control is a critical security process that regulates which entities can access specific data, resources, services and under what conditions. In Smart Hospitals, access control ensures that only authorized personnel or systems can interact with medical equipment, patient data, and hospital infrastructure. Access control is typically categorized into four main types [79]: (i) Role-Based Access Control (RBAC), which restricts access based on subject role; (ii) Discretionary Access Control (DAC), where resource owners grant access permissions; (iii) Mandatory Access Control (MAC), enforcing strict policy-based access often used in military or government contexts; and (iv) Attribute-Based Access Control (ABAC), which grants access based on specific attributes of users, devices, or environment, such as time or resource type. The complexity of access control in healthcare environments increases significantly due to the need for cross-domain data sharing. For example, in emergencies, patient data accessible only within Hospital A might need to be securely accessed by Hospital B to provide timely care. To address this challenge, the authors of [80] proposed a smart deduplication access control system that enables flexible, dynamic cross-domain data sharing by adapting access permissions based on the severity of emergencies through a break-glass access methodology. Additionally, the authors in [69] proposed SACS, a smart contract-based access control system for blockchain-enabled 6G healthcare networks. SACS incorporates multiple security attributes and has undergone formal verification and informal security analysis, demonstrating improved security, efficiency, and functionality compared to existing schemes.

With the emergence of 6G networks, characterized by heterogeneous devices and multi-connectivity, dynamic and context-aware access control mechanisms become essential to ensure secure and seamless data sharing while mitigating security risks. For instance, the authors in [81] introduced a software-defined ZTA that enables collaborative defense against network threats through trust-based access control, distributed identity management, and trust evaluation via Third-Party Security Services (TPSSs). This approach effectively mitigates threats such as worm-spreading malware and zero-day DDoS attacks. ZTA is a security approach to access control that enforces continuous verification and least-privilege access to resources, eliminating implicit trust and preventing unauthorized lateral movement within networks, operating on the principle of ‘never trust, always verify’ [82]. However, as the demands for rapid and scalable access control escalate, the limitations of traditional ZTA have become a concern. The authors of [83] propose a trusted ZTA by introducing trusted-based components, leading to significant improvements in performance, offering enhanced scalability and adaptability to modern-day challenges and simultaneously adding an additional layer of security to ZTA.

Based on our research, despite significant advancements in access control mechanisms, substantial challenges remain in designing robust and adaptive solutions capable of addressing the heterogeneous, dynamic, and high-demand environment of Smart Hospitals within 6G edge networks. Addressing these challenges requires multidisciplinary research efforts focused on scalable architectures, context-aware policies and formal verification to ensure secure, efficient, and trustworthy access control in next-generation Smart Hospital ecosystems.

#### 4.1.3. Anomaly Detection (AD)

AD involves identifying patterns in data that deviate from expected behavior. In the context of Intrusion Detection Systems (IDSs) for IoT, AD analyzes incoming traffic flows and flags deviations from normal behavior as potential anomalies, making them particularly effective for detecting unknown, novel attacks [84]. For example, AD-based IDSs demonstrate heightened efficacy in identifying DoS attacks and their variants [21].

Building on this foundation, several state-of-the-art approaches have been proposed to enhance AD performance specifically in healthcare IoMT and 6G network environments. For instance, the authors in [84] introduce a CLS-GAN-based IDS that uses two neural networks—a generator to create synthetic samples and a discriminator to classify inputs as real or fake—thereby enhancing threat detection accuracy. Meanwhile, FL frameworks have also gained prominence for their ability to preserve data privacy. The authors in [85] propose an FL-based AD system for IoT networks using multi-layer Gated Recurrent Units (GRUs) combined with ensemble techniques to improve prediction accuracy while preserving user data privacy. Evaluated on Modbus-based network datasets, this approach successfully detects MitM and DDoS attacks, outperforming non-FL intrusion detection methods.

Furthermore, the authors in [86] propose a privacy-preserving AD framework for IoMT in healthcare using a Federated Time Distributed (FEDTIMEDIS) LSTM approach. Deployed on edge cloudlets, this method shares only model gradients instead of raw patient data, thereby ensuring data privacy and model integrity while reducing computational and communication overhead. The approach facilitates easier collaboration among multiple healthcare organizations through disease-based grouping and hierarchical FL and has been evaluated on RPM use cases. Additionally, the authors of [87] present an AD framework for securing EHRs using isolation forest (iForest) and local outlier factor (LOF) algorithms to detect unusual access patterns. Their methodology, enhanced with classifiers such as Support Vector Machines (SVMs), decision trees, and random forests, achieves high accuracy and robustness in detecting contextual anomalies, including excessive access duration, atypical actions, or access beyond patient discharge, providing hospitals with an effective tool to monitor potential insider threats.

Expanding to 6G networks, the authors of [88] propose a hybrid ensemble-based IDS for 6G networks that leverages feature selection and ML techniques to detect a wide range of attacks, including DoS, DDoS, brute force, XSS, and SQL injection. Evaluated using multiple benchmark datasets such as CICDDOS2019, NSL-KDD, UNSW-NB2015, and CIC_IDS2017, their approach achieved high accuracy—up to 99.7%—with low false alarm rates. This demonstrates improved detection capabilities, especially for imbalanced and high-dimensional network traffic. The authors also emphasize the need for future development of adversarial-resilient models suitable for 6G and IoT environments. In addition, in a related research work focused on 6G-enabled metaverse healthcare analytics in IoT, the authors of [89] present a data stream AD (DS_AD) method that incorporates sliding window, change detection, and model update mechanisms within a Locality-Sensitive Hashing (LSH) framework based on iForest (LSHiForest). The sliding window technique manages infinite data streams, the LSH addresses data correlations, and the dynamic model update adapts to changing data distributions, optimized by change detection. Experiments on SMTP and HTTP datasets validate the feasibility of DS_AD for metaverse healthcare analytics. However, the study also highlights the critical importance of securing the cybertwin, a key component in 6G networks that is vulnerable to attacks and data leakage.

Finally, the authors of [90] propose a lightweight ML-based Anomaly-Based IDS (AIDS) for IoMT networks that monitors devices, gateways, and network traffic, achieving strong detection performance with low computational overhead. Similarly, the authors of [91] present a lightweight AIDS tailored for resource-constrained IoMT devices, using novelty and outlier detection algorithms to achieve strong intrusion detection with minimal CPU and memory usage. Additionally, the authors of [92] introduce an AIDS tailored for IoMT networks combining host-based and network-based monitoring integrated with ML-based detection at gateways to efficiently identify anomalies in IoMT environments.

Collectively, these advances highlight ongoing efforts to develop lightweight, efficient, and ML-driven intrusion detection mechanisms for securing the resource-constrained IoMT devices and gateways of 6G-enabled Smart Hospitals. However, based on our research, there remains a lack of comprehensive literature reviews addressing AD specifically in Smart Hospitals operating in 6G edge networks. This gap underscores a critical need for dedicated AD frameworks capable of accommodating the ultra-low-latency requirements, massive device connectivity, and highly distributed data processing intrinsic to next-generation healthcare ecosystems. Furthermore, AD for 6G-enabled Smart Hospitals must remain effective under highly dynamic traffic patterns driven by URLLC services, DTs, and massive IoMT connectivity, which significantly differ from traditional enterprise or even 5G traffic profiles.

#### 4.1.4. Security Information and Event Management (SIEM)

While most organizations implement security protocols and deploy antivirus software, 70% of cybersecurity specialists consider antivirus solutions alone insufficient, revealing significant gaps in incident management [93]. This underscores the need for comprehensive security solutions such as SIEM, which enable centralized collection, correlation, and automated response to security events. SIEM platforms aggregate, store, and correlate event data generated across the managed infrastructure, including inputs from IDSs, antivirus software, and firewalls. They analyze and correlate this diverse information to present integrated alert dashboards that facilitate threat management, incident investigation, and security compliance reporting [94].

However, current SIEM solutions face limitations, including constrained response intelligence, basic event correlation and analysis capabilities, limited storage capacity, heavy reliance on manual intervention, and underutilization of advanced features such as custom connectors and integration with external data sources [94]. Furthermore, the heterogeneous nature of IoMT devices and legacy systems, which may lack support for modern security protocols or logging standards, poses significant challenges for effective data collection and analysis in 6G-enabled Smart Hospitals. Addressing these challenges, the authors of [95] propose a novel SIEM system tailored for 6G edge networks that leverages decentralized and highly distributed AI algorithms for event correlation, threat detection and system protection. Given the sensitive and dynamic environment of Smart Hospitals within 6G networks, it is critical to employ modern SIEM systems capable of monitoring user activity, system and network logs, real-time nodes, and network traffic.

Finally, it is important to note that further research is required to enhance SIEM capabilities within Smart Hospitals operating in 6G edge environments. Future efforts should focus on advancing real-time threat detection, scalable and efficient event correlation, and adaptive response mechanisms specifically designed to meet the unique challenges and critical service requirements of healthcare infrastructures. This is especially important in 6G edge environments, where logs and telemetry originate from a much larger number of heterogeneous edge nodes, IoMT devices, and AI components, and where incident visibility must be maintained without violating latency and privacy constraints.

#### 4.1.5. Security Orchestration, Automation, and Response (SOAR)

SOAR systems extend beyond the capabilities of SIEM by automating and coordinating responses across the entire incident lifecycle, covering identification, containment, eradication, and recovery phases [96]. By integrating and automating security operations, SOAR platforms reduce the workload on Security Operations Centers (SOCs), thereby improving detection accuracy and response times. Although vendors have begun leveraging AI/ML technologies, fully end-to-end AI/ML-powered SOAR systems remain at an early stage. Future advancements are expected in areas such as deep reinforcement learning and enhanced interoperability across diverse security tools [96].

Given the increasingly interconnected nature of modern environments, especially in healthcare, the authors in [97] define the Internet of Blended Environment (IoBE) as a convergence of multiple systems and the Blended Threats (BTs) as complex security threats targeting these integrated threat surfaces. These threats demand substantial manpower and time for effective detection, analysis, and response. To address this challenge, the authors of [97] introduce the concept of Collaborative Units of Blended Environment (CUBE), which dynamically adapts according to the specific IoBE and associated threats. They propose a SOAR-CUBE architecture that automates response processes to BTs with minimal human intervention, automating the SOAR workflow in complex environments.

Moreover, a systematic review in [98] characterizes security orchestration as the unification, orchestration, and automation of security tools and workflows, emphasizing the urgent need for standardized evaluation metrics, reference architectures, and privacy-preserving mechanisms to facilitate broader practical adoption. These orchestration capabilities will be particularly valuable in Smart Hospitals operating within 6G edge networks, where diverse medical IoT systems have to be securely integrated, managed, and coordinated in real time.

In conclusion, our research identifies a clear gap: there is currently no comprehensive literature review focused specifically on SOAR systems tailored for 6G edge networks and Smart Hospitals. Bridging this gap is crucial for developing SOAR solutions that meet the stringent security, operational, and interoperability demands of Smart Hospitals in the 6G era. In particular, SOAR playbooks for 6G-enabled Smart Hospitals need to coordinate responses across clinical applications, edge analytics, and networking functions such as slicing and D2D communication, so that automated actions do not inadvertently disrupt time-critical medical services.

#### 4.1.6. Incident Response Systems (IRSs)

IRSs provide a structured framework for detecting, managing, and responding to security incidents, with SIEM and SOAR systems acting as key enablers. IRSs consist of the following key stages: (i) Preparation, which involves establishing policies, tools, and training; (ii) Detection, focused on identifying anomalies and potential threats; (iii) Analysis, which validates detection results and assesses the scope of incidents; (iv) Containment, which isolates affected nodes or systems to prevent propagation; (v) Eradication, which removes the root cause of the attack; (vi) Recovery, which restores system functionality and applies necessary patches; and (vii) Post-incident activities, including documentation of lessons learned and implementation of improved defenses [54,93,99].

The authors of [54] emphasize the importance of proactive threat management by integrating Cyber Threat Intelligence (CTI) into the Incident Response (IR) process. CTI provides actionable information regarding attack vectors, threat actors, impacted entities, and recommended responses, enabling more effective incident handling. Equally important, the integration of AI technologies in IRS has proven to drastically accelerate response times and improve adaptability to evolving threat landscapes [93].

Concluding, our research identifies a lack of comprehensive studies focusing specifically on IRSs in the context of 6G edge networks. This gap reveals a critical need to tailor IR frameworks to address the ultra-low latency, massive IoT connectivity, and distributed nature characteristic of 6G-enabled Smart Hospitals, ensuring security measures can meet their unique operational demands. Consequently, incident response in this setting must consider 6G-specific dependencies, including the tight coupling between edge-hosted functions, AI-driven orchestration, and ultra-reliable clinical sessions, when defining containment and recovery strategies.

### 4.2. Privacy-Preserving Technologies

In this section, we discuss privacy-preserving technologies relevant to Smart Hospitals operating within 6G edge network environments. Based on our literature review, the most frequently cited and adopted technologies in this context include FL, Split-FL (SFL), Compressive Sensing (CS), DP, Homomorphic Encryption (HE), and Secure Multi-Party Computation (SMPC). These technologies collectively address the stringent privacy requirements imposed by the sensitive nature of healthcare data, the massive connectivity of IoMT devices, and the low-latency demands characteristic of 6G-enabled healthcare systems.

#### 4.2.1. Federated Learning (FL)

**Figure 3:**
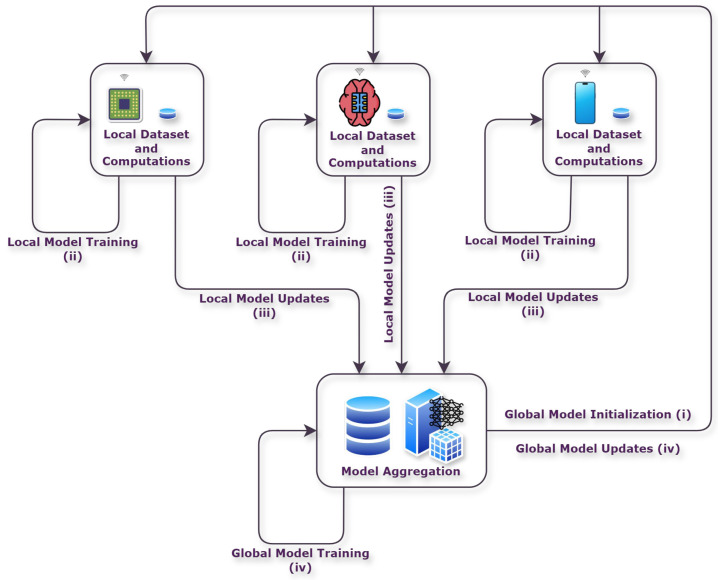
FL life cycle.

FL is a decentralized ML paradigm enabling multiple participants to collaboratively train a global model without sharing raw data. Instead of aggregating data centrally, each participant trains a local model on their private dataset and shares only model updates, such as gradients or parameters, with a central aggregator. This approach enhances data privacy, reduces communication overhead, and mitigates the risks of data leakage, while still enabling the development of accurate and scalable AI models. FL can be implemented in hierarchical or fully decentralized architectures, playing a crucial role in ensuring data security and privacy by keeping data close to the user within distributed 6G edge environments [21,39,100]. The typical FL process generally involves the following four main steps: (i) global model initialization by a central server initializing and distributing the global model to all or selected participating clients; (ii) local training where each client trains the model locally on its own data; (iii) update sharing where clients transmit only their model updates (gradients or parameters) to the central server, without exposing raw data; and (iv) aggregation and redistribution where the server aggregates the updates to form an improved global model for subsequent rounds. In particular, FL has the following life cycle as depicted in Figure 3.

FL has garnered significant attention for its application in smart healthcare, contributing to disease classification, medical diagnosis, pandemic management, drug discovery, medical device monitoring, medical image processing, RPM, and EHR management. These applications benefit from FL’s privacy-preserving features that address critical concerns regarding sensitive medical data [26,36,46,100,101,102,103,104,105,106,107,108,109]. For example, the authors of [40] propose a privacy-preserving FL framework using Secure Multi-Party Computation (SMPC)-based encrypted model aggregation for 6G-enabled IoMT environments. This framework ensures hospital models remain confidential during aggregation, supports secure inference, leverages edge computing for reliable connectivity, and achieves high prediction accuracy.

Nevertheless, FL faces specific vulnerabilities such as free-rider attacks, where malicious clients exploit the global model without contributing meaningful updates, ultimately degrading system performance [69]. Additional challenges, detailed in [46,100,103,105,106,108] include susceptibility to poisoning attacks, where adversaries inject malicious data to corrupt the global model, as well as fairness issues arising from unbalanced client data, handling heterogeneous and potentially low-quality datasets, scalability concerns for large numbers of clients or devices, efficient hyperparameter optimization, communication efficiency, and the establishment of standardized benchmarks and regulatory frameworks to ensure secure, robust deployment in healthcare IoMT environments. These limitations underscore the need for continued research to refine FL approaches for the unique demands of 6G-enabled Smart Hospitals, balancing privacy, scalability, and computational efficiency. Additionally, FL frameworks for Smart Hospitals must be co-designed with 6G resource management and slicing mechanisms so that model training and inference traffic does not interfere with, and is not starved by, latency-critical clinical communications.

#### 4.2.2. Split-FL (SFL)

SFL addresses certain limitations inherent in conventional FL by partitioning the model training process between clients and servers. Instead of clients sharing complete model updates, SFL restricts communication to the outputs of a designated intermediate layer, known as the cut layer, and their corresponding gradients. This division enhances data privacy by minimizing raw data and full model exposure, while also reducing computational overhead on client devices [110]. In addition, the authors of [111] review the application of SFL in 6G edge networks, highlighting its potential to reduce training times, preserve both data and model privacy, and balance computational workloads efficiently between clients and servers. While still in early development stages, SFL shows strong potential to improve the reliability and performance of future 6G edge networks. However, challenges such as limited availability of diverse datasets and concerns regarding system scalability remain open research directions. In 6G-enabled Smart Hospitals, SFL is especially attractive because it can be tightly integrated with edge-slicing and resource management, allowing computationally intensive parts of the model to reside on powerful edge or control nodes while keeping latency-sensitive and privacy-critical components close to medical devices and clinical workflows.

#### 4.2.3. Compressive (Or Compressed) Sensing (CS)

CS is a signal processing technique that simultaneously acquires and compresses data by exploiting the inherent sparsity and correlations in natural signals. This methodology enables accurate reconstruction of signals from fewer samples than traditionally required by the Nyquist criterion, significantly reducing sampling and transmission costs while supporting low-energy, privacy-preserving, and authenticated data acquisition, especially when integrated with edge computing technologies [112,113].

CS finds numerous applications in healthcare, as highlighted by [112], including wearable medical devices, medical imaging, electrocardiogram (ECG) and electroencephalography (EEG) monitoring, blood pressure and glucose level measurements, and biometric systems. By optimizing sensor energy consumption through CS-based techniques, the lifespan of such devices is substantially extended, while privacy is enhanced via embedded CS-enabled encryption methods. Extending these benefits, the authors of [114] propose a low-cost, privacy-preserving data sampling framework incorporating a two-layer chaotic encryption: chaotic encryption control during sampling, and chaotic permutation-diffusion after the sampling. This approach ensures secure data acquisition with minimal computational overhead, robust image reconstruction, and resistance to various attack vectors, demonstrating its suitability for protecting sensitive medical imaging data. Beyond healthcare, CS is recognized within 6G and edge computing literature as a key enabling technology for privacy-preserving data transmission in distributed environments, reinforcing its essential role in 6G-enabled Smart Hospital scenarios [39,115,116].

In conclusion, while CS exhibits substantial promise and has been validated in various IoT and 6G contexts, additional research is still needed to address challenges related to its practical deployment, optimization, and integration with smart healthcare systems operating over 6G networks. In the context of 6G-enabled Smart Hospitals, CS therefore serves a dual role: it reduces bandwidth and energy consumption on dense IoMT deployments, and it provides an additional layer of obfuscation for sensitive clinical signals in transit between sensing devices, edge nodes, and higher-layer analytics.

#### 4.2.4. Differential Privacy (DP)

DP is a mathematical framework designed to protect individual privacy during data analysis and ML tasks. It has emerged as a leading standard for privacy preservation and is expected to see widespread adoption in 6G networks for managing sensitive data queries. DP ensures that the results returned from data processing are carefully perturbed to prevent the leakage of privacy-sensitive information, thereby safeguarding individual data records while maintaining overall data utility [84,117]. A recent survey in [118] examines DP’s application in healthcare and medical systems, highlighting its use in real-time health data streams, EHR, and user-generated health surveys. The integration of DP with ML enables privacy-preserving analytics on sensitive medical data, though the demand remains high for lightweight DP mechanisms tailored for resource-constrained wearable devices. The survey also identifies the need for expanded research to broaden DP’s scope and enhance its practical deployment in healthcare.

Our literature review indicates a pressing need for focused investigations into the implementation of DP specifically within Smart Hospitals operating across 6G edge networks. This is critical for addressing the dual challenges posed by real-time processing of sensitive health data and the limited computational capabilities of IoMT devices in these environments. For 6G-enabled Smart Hospitals, DP must be carefully tuned to balance strong formal privacy guarantees with the accuracy and responsiveness requirements of real-time clinical analytics and network control.

#### 4.2.5. Homomorphic Encryption (HE)

HE is an advanced cryptographic technique that enables computations to be performed directly on encrypted data without requiring decryption, thus keeping sensitive information confidential throughout processing. According to [21], HE plays a critical role in mitigating privacy issues related to AI applications, which are central to Smart Hospitals operating within 6G edge networks. Specifically, HE effectively defends against poisoning and multiple types of ML model attacks, preventing unauthorized disclosure of private information.

The digitization of patient records has improved healthcare delivery and reduced costs; however, it also introduces significant security concerns due to the sensitive nature of EHRs [119]. HE addresses these concerns by enabling secure healthcare applications such as disease detection and privacy-preserving query generation over encrypted data. Furthermore, the protocol proposed in [120] can be applied to 6G-enabled Smart Hospitals with extensive IoMT deployments. By offloading computation to edge servers while preserving data privacy through HE, it ensures secure patient data processing, reduces latency, and facilitates real-time verification of results, satisfying the stringent privacy and performance requirements of 6G-enabled healthcare environments. Further extending HE applications, the authors of [121] highlight its role in safeguarding EHRs, healthcare databases, and secure medical data processing within cloud-based infrastructures. HE enables advanced analytics and collaborative healthcare services while maintaining robust protection of sensitive patient information.

Despite its advantages, the application of HE in 6G-enabled Smart Hospitals requires further investigation to optimize its integration with resource-constrained IoMT devices and edge computing infrastructures. Such research is essential to develop secure, privacy-preserving mechanisms that efficiently support large-scale, distributed processing of sensitive medical data in next-generation healthcare systems. In 6G-enabled Smart Hospital environments, HE is particularly relevant for offloading sensitive computations to edge or cloud resources without revealing raw PHI, but schemes must be optimized to meet stringent latency and throughput constraints imposed by URLLC and continuous monitoring applications.

#### 4.2.6. Secure Multi-Party Computation (SMPC)

SMPC is a cryptographic technique that enables multiple parties to collaboratively compute a function over their private inputs without revealing those inputs to each other. HE often serves as a foundational building block within SMPC schemes. In the context of 6G networks, SMPC is pivotal for privacy-preserving processing of sensitive data, including security incident analysis, by leveraging trusted execution environments on end devices to enhance flexibility in non-public network scenarios [113]. Given the increasing sophistication of attacks targeting both user and control planes in 6G, SMPC-based protocols combined with strengthened control plane robustness are essential to guarantee end-to-end confidentiality and integrity.

Additionally, SMPC plays a critical role in privacy-preserving ML tasks, which are fundamental to Smart Hospitals operating over 6G edge networks. The literature underscores the importance of balancing privacy preservation with implementation efficiency to maintain high data quality and system performance, especially in sensitive sectors such as healthcare and finance [114]. For example, the authors in [122] propose a privacy-preserving, self-serviced medical diagnosis scheme based on SMPC, facilitating confidential data exchange between medical data owners and hospitals, improving treatment accuracy, and reducing hospital computational overheads. Moreover, the authors of [123] present CRYPTEN, a software framework utilizing SMPC in ML tasks such as text classification, speech recognition, and image classification, all relevant technologies for Smart Hospital services.

Finally, comprehensive reviews, such as that in [124], outline SMPC theories, design methodologies, and applications, including secure genomic computation and privacy-preserving ML, while highlighting that practical implementations and addressing outstanding challenges remain active areas of research. Despite SMPC’s established status as a robust privacy-preserving mechanism, further focused research and real-world implementation are necessary to tailor SMPC methods to the unique privacy demands of Smart Hospitals within 6G edge network environments, thereby mitigating associated risks and optimizing usability. When deployed in 6G-enabled Smart Hospitals, SMPC can enable collaborative analytics and cross-institutional learning over distributed PHI without exposing raw data, but protocols must be engineered to remain efficient under high device counts, dynamic edge participation, and the tight timing budgets of critical healthcare services.

### 4.3. AI Model Hardening for Security Mechanisms and Privacy-Preserving Technologies

To mitigate the highly specialized AI threats identified in Section 3.1, such as data poisoning and evasion attacks, 6G Smart Hospitals must implement rigorous AI model hardening techniques directly at the edge. A primary security mechanism, in this context, is Adversarial Training, which involves intentionally injecting generated adversarial examples, such as mathematically manipulated medical images or perturbed sensor readings, into the local model’s training dataset. By exposing AI models to these specific evasion vectors during the training phase, the algorithm learns to identify and isolate malicious inputs. This significantly increases robustness, ensuring the system maintains a high diagnostic accuracy and operational integrity during real-time clinical inference [44,45,47].

Furthermore, to counter synthetic adversarial data generated by GenAI, defensive Generative Adversarial Networks (GANs) can also be deployed [125]. By utilizing GANs to proactively simulate evasion tactics during training, healthcare IoT models can effectively safeguard themselves against emerging automated attacks. For instance, the authors of [126] propose a defensive GAN architecture that neutralizes adversarial exploits within medical IoT environments, while achieving an ultra-low detection latency of approximately 82 milliseconds, making it highly suitable for 6G edge deployment.

### 4.4. Comparative Analysis and Critical Synthesis

To provide a holistic view of security mechanisms and privacy-preserving technologies for 6G-enabled Smart Hospitals, outlined in the left-hand panels of Figure 1, Table 2 and Table 3 present a comparative performance analysis. The evaluation considers key operational metrics, including computational cost, communication cost, security/privacy strength, strengths, weaknesses, and their adaptability to 6G edge environments. The qualitative ratings (Low, Moderate, High, and Very High) are based on a consistent scoring rationale that reflects the architectural and performance constraints of 6G-enabled IoMT environments. For computational and communication cost, Low denotes lightweight and bandwidth-efficient mechanisms that satisfy the low-latency requirements of real-time clinical workflows (e.g., CS, which reduces communication overhead by exploiting signal sparsity to enable efficient, low-latency data transmission [112,113]), Moderate denotes mechanisms requiring localized processing or periodic data exchange (e.g., SFL, which require localized model training and periodic parameter exchange among distributed edge nodes [111]), and High denotes resource-intensive mechanisms that require substantial bandwidth, continuous synchronization, or cloud-level offloading (e.g., SIEM, which continuously collect and analyze large volumes of security events, often requiring cloud-level resources to handle the processing load [94]). For security and privacy strength, Moderate denotes baseline protection against conventional threats (e.g., IRSs, which provide structured defense against known threats but remain primarily reactive to emerging attacks [54]), High denotes strong resilience against advanced attacks, including zero-day and lateral movement attacks (e.g., Software-Defined ZTAs in 6G, which dynamically restrict lateral movement and improve resilience against zero-day attacks in 6G environments [81]), and Very High denotes technologies that provide mathematically proven cryptographic guarantees despite their higher computational and communication overhead (e.g., SMPC, which enables collaborative computation while keeping each party’s data private through cryptographic guarantees [124]).

While the proposed mitigation mechanisms offer robust theoretical defenses for 6G-enabled Smart Hospitals, a critical analysis reveals a trade-off between security strength and operational overhead.

As detailed in Table 2, security mechanisms such as AI Model Hardening, Access Control, and AD are highly compatible with the massive connectivity of 6G, effectively preventing lateral threat movement and detecting zero-day attacks at the edge. On the other hand, traditional centralized orchestration tools such as SIEM and SOAR are not designed to operate at the same ultra-low-latency timescales as edge-native controls, and they often rely on human-in-the-loop workflows. As a result, they are better suited for centralized visibility, correlation, and coordinated response, while real-time mitigation at the 6G edge must be handled by lightweight, locally deployed mechanisms.

Additionally, a similar challenge applies to the privacy-preserving technologies outlined in Table 3. HE and SMPC provide robust privacy preservation; however, their high computational and communication costs render them impractical for direct deployment on resource-constrained medical wearables or within ultra-low-latency IoMT networks. Instead, they are better positioned as backend or edge/cloud-side services supporting high-assurance analytics and inter-institutional collaboration. By contrast, approaches such as FL and SFL offer a more practical balance for 6G edge environments by keeping sensitive data localized while distributing the computational burden across edge and cloud resources. Nevertheless, these ML-driven mechanisms introduce new attack vectors, such as data poisoning and free-rider attacks, which demand specialized cryptographic protection as a complementary level of defense.

Concluding, the security and privacy of 6G-enabled Smart Hospitals cannot rely on a single isolated mechanism; instead, they require a hybrid, decentralized architecture. Such an architecture should seamlessly map the mechanisms introduced in Figure 1, combining lightweight privacy-preserving technologies, such as CS, at the Intelligent Sensing Layer with AI-driven, automated threat detection, such as AD, embedded directly within the Intelligent Edge Layer to deliver real-time, low-latency clinical protection.

## 5. Conclusions and Future Research Roadmap

### 5.1. Conclusions and Critical Insights

This paper has shown that the security and privacy of 6G-enabled Smart Hospitals cannot be achieved through isolated mechanisms or traditional perimeter-oriented defenses alone. The literature indicates that security mechanisms such as Authentication, Access Control, AD, and AI Model Hardening are essential for protecting distributed clinical infrastructures, while privacy-preserving technologies such as FL, SFL, CS, and DP help reduce exposure of sensitive medical data. At the same time, a critical insight emerging from the review is the persistent trade-off between strong protection guarantees and the operational constraints of real-time healthcare environments, especially in terms of latency, computation, and communication overhead.

Another important conclusion is that centralized tools such as SIEM and SOAR remain highly valuable for global visibility, correlation, compliance, and coordinated response, but they must increasingly be complemented by edge-native and AI-assisted defenses capable of operating at the speed and scale expected in 6G environments. Similarly, high-assurance privacy-preserving technologies such as HE and SMPC provide strong confidentiality guarantees, but their direct use in ultra-constrained wearables and latency-sensitive IoMT workflows remains limited. Consequently, the most viable path forward is a hybrid and decentralized architecture that combines lightweight protection at the Intelligent Sensing Layer, adaptive security controls at the Intelligent Edge Layer, and coordinated trust management across higher architectural layers.

### 5.2. Future Research Roadmap and Open Questions

A systematic research roadmap for secure 6G-enabled Smart Hospitals should concentrate on three main priorities: (i) protecting ultra-constrained medical devices, (ii) hardening and stabilizing distributed AI, and (iii) enabling security operations that run natively at the network edge. These priorities arise from the key gap in current work, where security and privacy solutions are typically investigated in isolation, even though real Smart Hospital deployments will need integrated defenses that simultaneously meet security, privacy, reliability, and clinical safety requirements.

The first priority is to protect resource-constrained medical devices within the Intelligent Sensing Layer, including implantable devices, wearables, and bedside IoMT nodes. Future work should develop and systematically evaluate lightweight cryptography, physical-layer authentication, and patient-aware authentication schemes that operate within strict energy, memory, and latency constraints. A closely related open question is how to securely manage the coexistence of clinical IoMT and unmanaged personal devices, such as smartphones and mixed-reality headsets, which may create shadow-network attack surfaces in densely connected 6G hospital environments.

The second priority is resilient distributed AI across the Intelligent Edge and Intelligent Control Layers. FL and SFL remain promising for privacy-preserving analytics, but they still face open issues related to non-IID data, unstable connectivity, free-rider behavior, and poisoning attacks. In parallel, AI models supporting diagnostics, monitoring, and digital twins require stronger hardening against evasion, extraction, and synchronization attacks. Future research should therefore prioritize elastic FL protocols, robust aggregation strategies, adversarial training pipelines, runtime verification of digital twins, and explainable AI mechanisms that jointly preserve technical resilience and clinical trust [127,128].

The third priority is the development of distributed and adaptive security operations for 6G-enabled Smart Hospital infrastructures. Zero-trust principles are increasingly viewed as essential for 5G/6G systems because static trust assumptions are incompatible with highly dynamic and heterogeneous networked environments. However, important open questions remain about how to design clinically safe zero-trust policies, how to automate trust evaluation at scale, and how to integrate SIEM, SOAR, and incident response into low-latency edge workflows. Addressing these questions will require realistic testbeds, formal evaluation metrics, and close collaboration among cybersecurity researchers, clinicians, biomedical engineers, and regulators. Beyond these three domains, future work must also prepare Smart Hospital infrastructures for quantum-era threats. Large-scale quantum computers are expected to break many currently deployed public-key schemes, directly affecting authentication and key management across IoMT, edge, and backbone networks. This calls for the gradual design, evaluation, and integration of quantum-safe (post-quantum) cryptographic primitives and protocols that remain feasible on resource-constrained medical devices and do not violate the stringent latency and energy requirements of 6G-enabled clinical workflows.

Finally, future progress will require a shift from theoretical or simulation-based proposals to security frameworks that are validated, interoperable, and explicitly aligned with clinical practice. This shift depends on the creation of benchmark datasets, shared threat models, and experimental Smart Hospital testbeds that accurately capture real operational constraints. Such efforts are necessary to transform 6G-enabled Smart Hospital security from a promising research vision into a deployable, trustworthy reality for everyday healthcare. By framing security and privacy threats, mitigation mechanisms, and open research problems explicitly in relation to 6G edge network characteristics, the work aims to guide future efforts towards solutions that are not only theoretically sound but also practically deployable in next-generation Smart Hospital environments.

## Figures and Tables

**Table 1:** Summary of security threats in 6G-enabled Smart Hospitals, showing primary (✓) and secondary (✕) targets, main attack vectors, and compromised security goals.

Threat	Target						Primary Attack Vector	Compromised Security Goal(s)
	AI	Network		Devices		Human-Centric		
		Computer	IoMT	Medical	Personal			
Poisoning (Data injection, Data manipulation, Logic corruption)	✓						Malicious Data/Code Injection (Training Phase)	Integrity, Availability
Membership (& Reverse) inference	✓						Statistical Query Analysis	Confidentiality
Model inference (Model Extraction, Model Inversion)	✓						API Querying/Output Analysis	Confidentiality
Evasion	✓						Adversarial Sample Generation (Inference Phase)	Integrity
Brute Force		✓					Automated Credential Guessing	Confidentiality, Authenticity
Routing		✓					Network Path Manipulation/Misdirection	Availability, Integrity
SQL Injection		✓					Database Query Manipulation	Confidentiality, Integrity
Byzantine		✓	✓				Malicious Node Coordination/False Data Injection	Integrity, Availability
Shilling		✓	✓				Fake Profile/Malicious Rating Generation	Integrity
Desynchronization		✓	✓				Timing/Synchronization Protocol Disruption	Availability, Integrity
Node Injection		✓	✓				Unauthorized Device Addition to Network	Authenticity, Integrity
Node Subversion		✓	✓				Device Compromise/Takeover	Confidentiality, Integrity, Availability
Sybil		✓	✓				Forged Identity/Multiple Node Generation	Authenticity, Integrity
XSS		✓	✓				Malicious Script Injection (Application Layer)	Confidentiality, Integrity
Cookie Manipulation		✓			✕		Session Data Alteration	Authenticity, Confidentiality
Malware (Ransomware, trojans, spyware, viruses)		✓	✓	✕	✕		Malicious Payload Execution	Confidentiality, Integrity, Availability
DoS (Wormhole, Blackhole, Collision, Congestion, Overwhelm, Amplification, HELLO flood, Jamming)		✓	✓	✕	✕		Volumetric Traffic Flooding/Signal Interference	Availability
Impersonation		✓	✓			✕	Identity Theft/Credential Reuse	Authenticity, Confidentiality
Masquerading		✓	✓			✕	Deceptive Identity Presentation	Authenticity, Integrity
Forgery (Spoofing (IP/DNS))		✓	✓			✕	Network Protocol/Packet Falsification	Authenticity, Integrity
Eavesdropping (Sniffing, MitM (Replay), Traffic Analysis, Session Hijacking (Parallel Session))		✓	✓	✕	✕	✕	Wireless Signal/Network Traffic Interception	Confidentiality
DDoS				✓	✕		Distributed Traffic Overwhelm	Availability
IoT-botnet				✓	✕		Coordinated Malware Execution via Compromised Nodes	Availability, Integrity
Battery Drainage				✓	✕		Resource Exhaustion (Sleep Deprivation Attacks)	Availability
Energy Drainage				✓	✕		Continuous Protocol Polling/Processing Overload	Availability
Firmware Modification				✓	✕		Unauthorized Code Flashing/Overwriting	Integrity, Availability
Tampering				✓	✕		Physical Hardware Manipulation	Integrity, Availability
Device Cloning/Replication		✕	✕	✓	✕		Hardware or Cryptographic Key Duplication	Authenticity, Integrity
Key Logger						✓	Keystroke Interception (Software or Hardware)	Confidentiality
Phishing						✓	Social Engineering/Psychological Manipulation	Confidentiality, Authenticity
Digital Twin Manipulation	✓	✕	✕	✕			Real-Time Data Alteration/Synchronization Disruption	Integrity, Availability
Generative AI Exploitation	✓	✕				✓	Automated Vulnerability Probing/Synthetic Data Generation	Confidentiality, Integrity, Authenticity
Quantum-enabled cryptanalytic attacks	✕	✓	✓	✓	✕	✕	Cryptographic key	Confidentiality, Integrity, Authenticity

**Table 2:** Comparative Analysis of Security Mechanisms in 6G Smart Hospitals.

Mechanism	Computational Cost	Communication Cost	Security Strength	Strengths	Weaknesses	6G Edge Adaptability
Authentication	Low	Low	High	Fast, secure, prevents unauthorized access; lightweight variations exist for edge devices.	Traditional methods struggle with heterogeneous devices; blockchain-based authentication demands high consensus overhead.	High. Crucial for seamless multi-connectivity and distributed edge environments.
Access Control	Moderate	Moderate	High	Facilitates dynamic, cross-domain sharing; zero-trust eliminates lateral threat movement.	Traditional ZTA lacks rapid scalability; complex policy management in high-demand environments.	High. Software-defined and smart contract-based access fits the distributed 6G topology well.
AD	Low	Low	High	Highly effective at detecting zero-day and novel attacks; handles infinite data streams.	Requires constant model updating; vulnerable to adversarial ML attacks; high false-positive rates if unoptimized.	Very High. Can be embedded directly within the edge layer for real-time protection.
SIEM	High	High	Moderate	Centralizes event collection and correlation; provides compliance reporting and threat dashboards.	Constrained response intelligence; high reliance on manual intervention; struggles with legacy IoMT logs.	Moderate. Requires integration with decentralized AI to handle the massive data volume of 6G networks.
SOAR	High	High	Moderate	Automates the incident lifecycle; heavily reduces SOC workload; adapts to BTs.	Fully AI-powered systems are still in their infancy; lacks standardized evaluation metrics.	Moderate. High potential for 6G orchestration, but requires further maturity to handle edge-speed automated responses.
IRS	Moderate	Moderate	Moderate	Provides a structured framework (Preparation to Recovery); integrates CTI.	Highly reactive unless paired with predictive AI; difficult to execute within the ultra-low latency constraints of 6G.	Moderate. Needs significant tailoring to operate effectively at the latency and distributed nature of 6G.

**Table 3:** Comparative Analysis of Privacy-Preserving Technologies in 6G Smart Hospitals.

Technology	Computational Cost	Communication Cost	Privacy Strength	Strengths	Weaknesses	6G Edge Adaptability
FL	High	Moderate	High	Keeps raw data local; scalable.	Susceptible to data poisoning and free-rider attacks; struggles with heterogeneous datasets.	High. Decentralized nature aligns perfectly with edge computing nodes.
SFL	Low	Moderate	High	Reduces client overhead; exposes only cut-layer outputs.	Limited availability of diverse datasets; open questions on global scalability.	High. Effectively balances workloads between resource-constrained IoMT and edge servers.
CS	Low	Low	Moderate	Simultaneous acquisition and compression; extends device battery life.	Practical deployment, optimization, and system integration remain open research challenges.	High. A key enabler for privacy-preserving data transmission in distributed environments.
DP	Moderate	Low	High	Protects individual records while maintaining overall data utility.	Requires focused investigations for practical edge processing implementation.	Moderate. Needs optimization for real-time IoT.
HE	High	High	Very High	Computes directly on encrypted data; mitigates poisoning attacks.	Highly incompatible with low-latency IoMT workflows without edge offloading.	Moderate. Impractical for wearables and requires offloading to edge servers.
SMPC	High	High	Very High	Collaborative computation without revealing private inputs.	Complex implementation; introduces significant computational and communication overhead.	Moderate. Highly secure but requires trusted execution environment.

## Data Availability

No new data was created in this study. Data sharing is not applicable to this article.
